# The psychosocial problems of families caring for relatives with mental illnesses and their coping strategies: a qualitative urban based study in Dar es Salaam, Tanzania

**DOI:** 10.1186/s12888-016-0857-y

**Published:** 2016-05-14

**Authors:** Masunga K. Iseselo, Lusajo Kajula, Khadija I. Yahya-Malima

**Affiliations:** Department of Clinical Nursing, Muhimbili University of Health & Allied Sciences (MUHAS), P.O.Box 65004, Dar es Salaam, Tanzania; Department of Psychiatry and Mental Health, Muhimbili University of Health & Allied Sciences (MUHAS), P.O.Box 65001, Dar es Salaam, Tanzania; Tanzania Commission for Science & Technology (COSTECH), Ali Hassan Mwinyi Road, Kijitonyama (Sayansi) COSTECH Building, P.O. Box 4302, Dar es Salaam, Tanzania

**Keywords:** Relatives, Mental illness, Psychosocial problems, Caregiver

## Abstract

**Background:**

Mental illness may cause a variety of psychosocial problems such as decreased quality of life of the patient’s family members as well as increased social distance for the patient and the family caring for the patient. Psychosocial challenges are enhanced by the stigma attached to mental illness, which is a problem affecting not only the patient but also the family as a whole. Coping mechanisms for dealing with mentally ill patients differ from one family to another for a variety of reasons.

The aim of the study was to determine the psychosocial problems of mental illness on the family including the coping strategies utilized by family members caring for a person with mental illness.

**Method:**

A qualitative study was conducted, involving four focus group discussions and 2 in-depth interviews of family members who were caring for patient with mental illness at Temeke Municipality, Dar es Salaam. Purposive sampling procedure was used to select participants for the study. Audio-recorded interviews in Swahili were conducted with all study participants. The recorded interview was transcribed and qualitative content thematic analysis was used to analyse the data.

**Results:**

Financial constraints, lack of social support, disruption of family functioning, stigma, discrimination, and patients’ disruptive behaviour emerged as the main themes in this study. Acceptance and religious practice emerged as the major coping strategies used by family members.

**Conclusion:**

Familial care for a person with mental illness has its advantages, yet it has multiple social and psychological challenges. Coping strategies and skills are important for the well-being of the caregiver and the patient. Addressing these psychosocial challenges requires a collaborative approach between the health care providers and government so that the needs of the family caregivers and those of the patients can be addressed accordingly.

## Background

Globally, it is estimated that 450 million people are affected by mental disorders at any one time. These include 121 million people with depression, 24 million with schizophrenia and 37 million with dementia [1]. Mental illness accounted for about 12.3 % of the global burden of disease in 2001 and it is estimated that by 2020 unipolar depressive disorders will be the second most important cause of disability [2]. The burden of caring for mentally ill patients falls on the family members who provide all necessary support.

Evidence from developing countries is scarce; some countries show a higher prevalence of psychiatric patients living with families. In Uganda for example, the Uganda National Health Statistics (UNHS) of 2005/06 reports that of all households with disabled members, 58 % had at least one person with a mental disorder. To date, Tanzania lacks established statistics on families affected by mental illness.

Most mentally ill patients normally consult traditional healers and the reasons for not attending health facilities still remains unclear [3]. Beliefs regarding the cause of mental illness may be one of the reasons for not seeking healthcare. Most family members view mental illness not as disease, but as a curse; a product of both witchcraft and evil spirits of which the patient himself is counted as the main contributor [4]. It is likely that mental illness is severely underreported resulting in many untreated patients. However, in all cases, family members bear much of the burden of the patient’s mental illness and this affects them psychologically and socially [5].

### Psychosocial problems

Mental illness may cause a variety of psychosocial problems such as decreased quality of life for the patient’s family members, as well as increased social distance for the patient and the family caring for the patient. The family members who care for relatives with mental illness report feeling stigmatized as a result of their association with the mentally ill [6].

Persons with serious mental illnesses often engage in behaviours that are frightening, troublesome, disruptive, or at least annoying, and many relatives are obliged to control, manage, or tolerate these behaviours [7]. Thus, psychiatric professionals often view the family members of a patient as people of support because they can act as informants regarding the patient and they can act as co-therapists at home [8]. The family members need to be in an optimal social and psychological state. It is reported that reduced function of one family member contributes to the burden of other members and this in turn leads to other family members assuming a critical attitude towards the patient [9]. Such criticism can in some cases lead to a relapse of the patient’s illness or to the family feeling overwhelmed by the patient’s disruptive behaviour [6, 10].

Social support is important for the wellbeing of the family affected by mental illness. A research finding reveals that families should assume major roles in supporting relatives with mental illness; and collaborative plans should include strategies to assist family members and consumers in dealing with stigma [11]. There is a relationship between caregivers’ social support and stigma associated with relative with mental illness. For example, in a study investigating the links between stigma, depressive symptoms and coping amongst caregivers, it was found that stigma may erode the morale of family caregivers and result in withdrawal from potential supporters [12]. This argument supports the situation of families affected by mental illness in Tanzania as many families with mentally ill members hide the patient to avoid stigma and being socially discriminated. It is not known what psychosocial problems affect family members caring for their relative with mental illness in Tanzania.

### Family coping and adaptation

Coping differs from one family to another for a variety of reasons. In developed countries, some researchers have emphasized coping as a key concept for the study of adaptation and mental health [13, 14]. However, the effects of age, duration of illness, living arrangements and other contextual factors on the coping styles of family caregivers, and on the recovery or rehabilitation of persons with mental illness are important factors to be considered [14].

In this case, family caregivers have to learn and understand the patient’s characteristics and behaviour. Coping with symptoms such as delusions, hallucinations, inappropriate behaviours, and violence may often require lengthy, complex, and distressing negotiations. Over-burdened caregivers employ less effective coping strategies, report more frequent physical and mental health problems and use services more often [15].

In the Tanzanian context, however, it is unclear how family members cope with the mental illness of their relatives.

It is important to know how the family struggles to cope with the stressful situation so as to be able to help the family with mental illness. This study examined the psychosocial problems and coping strategies of families living with a person with mental illness within Temeke Municipality, Dar es Salaam.

## Methods

### Study design

The study used an explorative qualitative approach. Information was obtained from individual families living with mentally ill persons regarding the problems and coping strategies utilized.

### Setting

The study was conducted in Dar es Salaam, which is the largest commercial city in Tanzania. The city has a population of more than four million and has diverse socio-cultural characteristics as a result of significant rural to urban migration. This region consists of a mixture of tribes mainly from southern Tanzania.

### Sample size and sampling procedure

Fourteen participants were recruited using purposive sampling on the clinic days at Temeke Municipal Hospital in the Outpatient Clinic of Mental Health and Drug Abuse. Family members escorting the patient with mental illness to the clinic and who met the criteria were asked to participate in the study. Temeke Municipal Hospital does not admit psychiatric patients; therefore those patients who need admission are referred to Muhimbili National Hospital, which is also situated within Dar es Salaam. Sampling was done to the point at which no new information was obtained from participants and redundancy was achieved, referred to as data saturation. During the interview we identified two participants who seemed to be reserved, and were not active participants. We interviewed them separately after the Focus Group Discussions in order to obtain their views and experiences.

### Selection criteria

Participants were selected according to the following criteria:

1. Caregiver living with relative who had been suffering from mental illness for more than 6 months.
2. Caregiver is 18 years or older
3. Caregiver is the main caretaker.

He/she was explained briefly the nature of the study before being asked to participate. All who agreed to participate were asked to provide contact information to decide on an appropriate day for the interview.

### Ethical approval

Ethical clearance was obtained from the Muhimbili University of Health and Allied Sciences Ethics and Publication Committee. Further permission and approval was obtained from the Temeke Municipal Medical Officer in charge. Before the interview started, the informants were given a full explanation of the data collection procedure. Each participant was fully informed and understood the nature of the study and voluntarily agreed to participate. The written consent form was read, understood, and signed by the participants. Those who were not able to write were requested to provide verbal consent. However, all were able to read and write and therefore signed the consent form. Participants were informed that they could continue to use the health service as usual. Confidentiality was guaranteed by maintaining anonymity of all caregivers who participated in the study. Freedom to withdraw from the study at any time was ensured.

### Data collection

Four focus group discussions (FGD) and two in-depth interviews were carried out due to the fact that more detailed information was needed in order to get the psychosocial problems and coping strategies of families caring for patients with mental illness. Data were collected through audio-taped interviews, which lasted for 55 to 60 min for focus groups discussions and 30 to 45 min for in-depth interviews. Four FGDs were conducted at the hospital. Each FGD consisted of 3–4 members. The aim was to get at least 6–8 people but it was not possible for a variety of reasons including problems with transportation There was no categorization of the group participants. Both sexes were mixed during the interview and they were from different areas of the Dar es Salaam region. The in-depth interviews were conducted in the participants’ home environments. This provided an opportunity for the researcher to observe the living situation of the caregiver and the patient. The participants in this category were identified during the interview as those who were not actively sharing their views in the group discussions. They were then asked to participate in an interview in their home environment.

Data collection was guided by the following five structured questions.

1. What is the experience of caring for your mentally ill relative?
2. What problems do you face when caring for the mentally ill relative?
3. How do other relatives help you to care for the mentally ill relative?
4. What are the attitudes and views of the people around you concerning caring for a person with mental illness?
5. How do you cope with such problems?

The structured questions were followed by specific probing questions in order to obtain more information or clarification. The discussions and interviews were conducted in Kiswahili, the national language which was easily understood by all informants. During group discussions, the principal investigator moderated the discussion while a research assistant was taking notes, operating the audio recorders and taking care of any interruptions. Observations of non-verbal reactions were collected during and after the discussion. Field notes were blended with the recorded data during analysis.

### Data analysis

Content thematic analysis was used in order to gain a deeper and clearer understanding and a formation of themes. Audiotaped focus group discussions and in-depth interviews were transcribed verbatim in the original Kiswahili language. The principal investigator transcribed the audiotaped data by typing directly into the computer using the Microsoft Word program. This helped the researcher to correlate the tone of the informants in the sentence with the feelings and emotions, which were of importance to the analysis. In the process of transcription, the informants’ words were captured as closely as possible. To make sure that the data were transcribed correctly, the transcripts were checked against the audiotape and then reviewed by a person who was also proficient in Kiswahili. Interview notes and ideas were jotted down and the transcripts were read several times so that the researcher could be immersed in the data.

The unit of analysis was the themes expressed in the text about social and psychological problems found in families caring for persons with mental illness. Iterative reading of the interview transcripts was done. Pens, highlighters and memos were used during analysis. Different colours were used to highlight the patterns in the text, which corresponded to the preconceived category derived from the study objectives. In both margins of the hard copies of the transcripts, the patterns were jotted down in a crystallised meaning (condensed meaning) which were then transferred to a different sheet of paper for further analysis. Memos were used to summarize the patterns of the condensed meaning before transferring to the master sheet. To identify the source of data in the memo and in the text, identification numbers of informants were used when transferring the condensed meaning to the master sheet, so that the source could be easily traced and accessed. On this sheet of paper, coding schemes were developed with the abstracted categories and themes. Different categories, condensed meaning units, or codes were compared for underlying meanings and relationship at the interpretation level, which formed the themes (Table 1).

**Table 1:** Some excerpts of coding scheme resulted in theme formation

Meaning unit	Condensed meaning unit	Code	Category	Theme
The problem which I get is that I can’t even chop onions in public, I always do that in the locked room, and all knives and everything are hidden in the cupboard	Doing thing in hidden places to avoid physical attack	Fearing physical attack	Fear of being attacked	Problems in Managing patient’s symptoms
There is a day he can really succeed to kill, if not him to kill, himself may be killed, because I can prevent when attempting to hurt me, unfortunately hurts himself.	Expressing fear of patient to kill or to be killed	Unpredictable patient’s behaviour	Patient’s safety	

Field notes were also analysed separately whereby the patterns and categories were compared to those from the FGDs and in-depth interviews.

The two lists of categories were generated and compared and adjustments were made to the words used in the labelling of the categories. Transcripts were read again alongside with the list of the categories to ensure that the interviews had been transcribed. All the transcripts were then coded according to the list of categories and the coded sections of the transcripts were collected and then sorted. Verbatim examples were used as evidence for each theme and categories.

## Results

### Characteristics of informants

A total of fourteen family caregivers (informants), whose age ranged from 35 to 60 years, were interviewed. Five informants were men and nine were female. Four informants identified as Christian and ten identified as Muslim. Most of the informants had gone to primary school and a few had received secondary level education. Five informants were housewives, that is, they were married with no source of income except that of their husbands while five participants were petty traders’ i.e. were doing small business (Table 2).

**Table 2:** Characteristics of informants

Charactristics	Frequency
Age	
25–34 years	0
35–44 years	4
45–54	6
55 and above	4
Gender	
Male	5
Female	9
Kinship	
Mother	8
Father	3
Sister	1
Brother	2
Level of education	
Primary	9
Secondary	3
Non educated	2
Religion	
Christian	4
Islamic	10
Occupational	
Employed	1
Not employed	5
Self employed	8

### Characteristics of patients

The informants who participated were caring for ill relatives with different types of mental illness including schizophrenia, bipolar disorders, autistic disorders and epilepsy with psychosis. The duration of relatives’ illness ranged from 7 months to 27 years. All the caregivers were living with the patient in the same household and most of them were parents.

## Themes identified

After analysis, seven main themes emerged from the data.

### Financial constraints

Financial constraints were a concern for almost all participants. They said they had limited time to work in order to earn money because most of their time was spent caring for their relative. The amount of money earned from limited working time was used to help care for the relative, such as buying bus fare to the hospital, purchasing medications, and other activities for the patient.

### Cost for transport

Most informants expressed concerns about financial strain caused by increased cost for patient care, which was coupled with decreased working hours. Most of the participants were living a considerable distance from the hospital, they had to travel either by taxi or by public transport for patient follow-up. This caused most caregivers to have multiple financial crises with the small amount of money they had. For psychotic patients, some used to take taxi but the easiest and most accessible transport was public buses (*daladala*). The most important concern was how to get the money for the transport. Some failed completely to come to the hospital for more than two or more months and other caregivers sometimes used their other relatives to collect the medication at the clinic as reported by one of the respondents caring for her elder sister with schizophrenia:

“*Like that day I sent my brother-in-law, and told him; take this patient’s register book because you are going there, I don’t have the bus fare for going to the hospital just to collect the medicine*.” (46 year old sister).

### Cost for medication

In addition to cost of transportation, it was revealed that shortage of medicine in the hospital compromised the treatment system of the patient. Medication was expressed as a major contributor to a patient’s improvement. For example, the informants whose ill relative used more than two types of medication were rarely getting the second medication from the hospital and consequently had to purchase it in private pharmacies. As a result of lack of money, they used only one type of medication due to the carer’s inability to buy the second drug. This was reported by an informant caring for his son with schizophrenia:

*“……and when you come the next month, you are told; again, we don’t have this type of drug, so you are given the only one, okay! Then the patient uses the one type of drug because no money to buy the second drug.” (*58 years old father).

The psychotropic medications are normally provided free of charge by the government hospital. However, not all are available in the government pharmacies and thus patients need to buy from private pharmacies. In this case patients reported that drugs were so expensive that those who managed to buy them had to sacrifice other household needs so that the patient could get the drug to prevent the disruptive behaviour:

*“My main problem is money to buy the drug. They (doctors) have changed the medication, just imagine, a single monthly dose now costs forty thousand shillings (40,000/=) and she uses three different types of drugs……we ought to abandon other household issues to buy the medication because if she does not use the drugs for two or three consecutive days, you find that no peace at home”. (*51 years old mother).

This theme occurred even in patients using one type of drug. Some informants expressed that even if the patient had one type of drug which was not available at the hospital, the patient had to wait until they get the money to buy it. This was expressed as a causative risk factor for frequent relapse of patients’ symptoms as a result of inappropriate dosage regimen.

The informants expressed that their relative’s disruptive behaviour needed to be prevented by regular use of medicine. Lack of money for transportation and medication for the patients was suggested to be the main impediment to the patient improvement.

### Disruption of family functioning

Family functioning is the ability of the family to continue with daily activities despite an internal or external threat. Most participants expressed disturbances in their normal routine as a result of having a mentally ill person in the household as explained below:

### Disrupted household routines

Disruption of household tasks and other responsibilities were important sources of distress revealed by many caregivers. Unpredictable patient symptoms seemed to be a distressing factor which limited caregivers’ time for other family responsibilities. Parents of patient with mental illness had multiple roles including caring for the patient as well as making sure that other family members were getting their needs met. One caregiver whose ill relative was totally dependent said that she devoted most of her time doing activities for the patient. This affected the family caregiver income and hence made life more difficult as expressed by one of the respondents:

*“So far, I am no longer doing any work, my work has been compromised by staying at home all the time caring that one (the ill daughter); no any more work to do. I have to give medication, making sure she is safe and my properties are safe”* (51 years old mother).

Another one added:

*“Really I am affected …….you ought to remain at home to protect him probably unanticipated catastrophe may occur. If you attempt to go out to do your work, you become anxious because he (the patient) may be violent.” (42 years old father).*

Most parent caregivers stated that it is their responsibility to care for the patient because it is their child who is suffering and they cannot give to anyone else. Because they have to work, some caregivers reported locking in the patient when they needed to go out for daily activities. One caregiver aged 59 years old caring for her son with autism expressed that, “even this time I am here; I have locked in my son in his room until I come back.” She insisted that the patient is totally dependent on her and that he is unable to express his needs. Although some informants said that they have other family members living in the same household, they contribute little to care of the patient.

### Lack of family harmony

Resilience in the family was also affected by the patient’s disruptive or by family members disagreeing about the management of the mentally ill relative. One caregiver expressed concern about her husband by saying:

*“...........He uses abusive language to my son because his is mentally ill but he does not show such behaviour to the children. We always quarrel for that reason*” (50 years old mother).

Other informants revealed that a frequent source of misunderstanding revolved around how to find solutions to the patient’s problems. It was revealed that one parent/member may seek out help from a traditional healer for treatment while the other may seek out spiritual treatment. However, most caregivers resolved to seek out professional treatment.

### Disruptive behaviuor of patients

#### Problems in managing patients’ symptoms

Participants had difficulties in managing patients’ symptoms. They expressed concern that there was no one else who would be able to handle the unpredictable behaviour of the patient. They were the only one who had learned how to handle the patients’ behaviour; therefore, they had to remain at home to protect the patient and other people from patient’s uncontrolled behaviour. The majority expressed fear of being attacked by the patient as well as concern for safety of the patient as elaborated below:

*“I have got many problems with my patient because, he is very strong and fat and his intention is to kill me and his father. The problem which I get is that I can’t even chop onions in public, I always do that in the locked room, and all knives and everything are hidden in the cupboard.” (*51 years old mother).

Some other informants expressed that they had to be modest and humble when talking with the patients. They revealed that some family members do not bother to learn about the patients’ behaviour and how to handle him or her when agitated. Thus leaving care of the patient to the main caregiver despite the dangers to that person.

### Patients’ safety

A good number of caregivers expressed concern about safety of the patients who at times aimed to attack and kill others or themselves as echoed by a 35 year old man caring for his young brother with paranoid schizophrenia:

*“………there is a day he will succeed to kill, if not to kill him, he may be killed, because I can prevent him from attempting to hurt me, unfortunately he can hurts himself.” (*35 years old brother).

Concern by family members for the safety of the mentally ill relative caused many to experience anxiety especially when the relatives get lost in the street or when they become disruptive and violent. Parents caring for epileptic patients stressed that when their ill children get out, they did not remember to return home. The caregivers had to take time and effort to look for the patient everywhere. Some patients were reported to get lost for several weeks.

*“That day I told him to go home alone, but he was lost for about a month, I mean he did not return home. After looking for him for two weeks, I found him in the social beach near a deep water level*” (56 years old father).

Participants reported that patients were more aggressive and violent when they do not take their medication and this was a common problem provided that some medications had to be bought at private pharmacies. Furthermore, family resilience was also disrupted when the patient interfered with normal family social life such as by preventing family members from engaging in basic activities such eating and watching television.

### Conflict with neighbours

The caregivers revealed that their relatives’ uncontrolled behaviour such as temper tantrums, shouting in public, insulting people and neighbours and hitting people had caused them to have difficulty in forming and maintaining good social relationships. Some caregivers had been blamed for their relative’s behaviour in court of law causing endless misunderstanding between family with the ill relative and the neighbourhood family. One informant reported that:

*“When you make jokes of him several times, then, he becomes aggressive suddenly attacks you even stabbing you if in access with a knife; I have been arrested by police about two times to answer cases.”* (42 years old father).

It was said that most people do not know the nature of the mental illness and think that the person was pretending. Most of the family members tried to illuminate that their ill relative is not pretending and that he/she is suffering from mental illness.

### Lack of social support

In this study many informants expressed lack of support from other people, both inside and outside the family. It was revealed that distance between other relatives and the caregivers increased as the patient’s symptoms increased. Ignorance of family members in regards to the nature of mental illness was said to be a contributing factor to lack of support as most of them thought that the illness was of short duration. For example, if the patient’s symptoms lasted longer than they expected, they gave up and withdrew their support. Others reported that the patient’s symptoms are caused by the parents trying to get wealthy by making their child mentally ill. One caregiver reported that ‘no relative will come to give you anything rather than advising you to go to the traditional healers.’ Financial or material assistance was mentioned to be very important but this was not always offered even though some have close relatives in good financial positions:

*“She (the patient) has many siblings here in Dar es Salaam, her brother, sister; both are workers but not even phoning to know how you are progressing there. ……..they are educated with cars, oh, no even phoning.”* (46 years old sister).

Participants acknowledged that family dynamics are changing from that of an extended family system to more of a nuclear family system where the welfare of a child is the sole responsibility of the parent. No one may take part in taking care of one’s’ child in health or in illness. A man aged 58 years old caring for his son with epileptic psychosis said:

*“When you get such problems bear in mind that it is yours; neither uncle, cousin nor whoever, according to the current situation will assist you in anyhow.”* (58 years old man).

Caregivers described feelings of helplessness related to the fact that no one else was willing to help them care for their child and that they had to persevere with caring problems as living with such patients need cooperation with other members of the families.

### Need of self-help groups

Some caregivers reported a desire for social support groups designed to improve the quality of life of mentally ill individuals. These social groups could be of any nature including but not limited to educational or religious in order to assist their ill relatives in socializing and thus relieve any distress caused by loneliness. Also they were concerned about the deteriorating cognitive function of their mentally ill relative and expressed a desire to find educational groups.

“*If we get such group which can help to socialize and assist such children and intermingle with those who are mentally stable, could greatly help them. …….because we toil to go here and there as a result we become weary in vain.”* (A 42 years old father).

Furthermore, it was also revealed that there is a need for caregiver support groups to address physical and psychological problems developed by caregivers themselves as a result of caring for their mentally ill relatives. Professional or social support was frequently mentioned by caregivers while venting about their emotional distress:

*“You become tired of everything; you wish to have new ideas or people to help or group to mix with for getting new challenges……..you find that we are also tired of thinking, tired of strength, at the same time running short of money.”* (A 40 years old mother).

Many informants suggested having this social group as a way to simultaneously improve the welfare of the patient and their own well-being. This was suggested as an alternative to admitting mentally ill relatives to mental health institutions, something some informants had seriously considered. Others suggested creating schools for mentally ill individuals with qualified teachers because some patients had improved their mental status but were unable or afraid to join public schools.

### Stigma and discrimination

Many caregivers described people around them having negative attitudes toward their mentally ill relative. Most informants reported that the relative was more stigmatized than the caregivers themselves and that when they hear or see their ill relative being ostracized, they feel guilty and are psychologically disturbed. However, the experience of societal stigma and discrimination is worsened by negative attitudes from close relatives towards the caregivers, thus creating an increased distance among them and the community at large. Negative attitudes from close relatives were explained to occur on different occasions such as when using public transportation and at other social gatherings. One participant whose ill relative had cannabis induced psychosis complained that his neighbours shunned his son from social events:

*“Because even if he sees them sitting and chatting, and if he decides to go there to sit with them, you find that they all stand up and start leaving away. They perceive him as insane and can’t chat with them*.” (38 years old mother).

Some highlighted that stigma was present among family members. This was apparent after the onset of illness. This seemed to be an unbearable burden to the caregiver as everything is left to him/her.

*“Stigma is present even among us, because, you find that initially, you will be with your family and the society in general, but when the problem happens, none of your relative will come to know your progress with the patient.” (*38 year old mother*).*

Stigma was said to be caused by lack of knowledge about the nature of mental illness. The caregivers expressed their belief that education regarding mental illness should be provided to people in order to prevent stigma and discrimination. Most caregivers had demonstrated interest in understanding the origins of mental illness and some had even obtained brochures from some mental health facilities with respect to their relative’s condition. Patients were said to be more stigmatized than the caregiver probably due to awkward behaviour. However, caregivers were blamed for causing the ill relative to be in that situation to begin with.

### Coping and adaptation

Coping and adaptation are one of the important aspects to be applied when caring for a patient with chronic mental illness. Different types of coping and adaptation were reported by the participants.

Acceptance and faith were two of the most frequently cited strategies for coping. Caregivers had learned to accept and reconcile the disability or deviant behaviour in the mentally ill relative so as to avoid the dissatisfaction and disappointment that could have resulted from the patient’s bizarre behaviour.

*“You need to accept, no way, that is your child, whether your relative, your family member. …….where would you ask help? That is your gift from Almighty God, you have to accept.” (58 years old father).*

Some family caregivers took a positive step by utilizing problem solving strategies to address their relatives’ psychological, emotional and practical needs. Most family members brought their ill relative to the hospital after consulting different areas especially from traditional healers.

*“So, it is the hospital services we are continuing with until now. It gives me hope and satisfaction I and the patient will never go to the traditional healer again”* (60 years old mother).

Other family caregivers sought religious support as the only means of hope and encouragement. They said that their religious practice gave them peace of mind and helped them to endure the caregiving situation. They believed that praying was also likely to reduce the suffering of their ill relative as well, thus making their faith indispensable to continued caregiving of the relative, irrespective of their distressing behaviour.

*“Whenever the aggressive behaviour of my son begins, I immediately enter into my room, lock the door, kneel down and pray for Almighty God; Oh God! Help me to touch the Cross until my time of death*!” (50 years old mother).

Some caregivers displayed signs of despair due to the difficult life situation caused by the patient’s critical demands coupled with unrealistic daily income. They had no means of coping or adapting to manage their situation either due to persistent bizarre behaviour by the patient or their own lack of energy. However, they still had hopes due to their faith;

*“That’s why sometimes you speak rudely to me, and I answer rudely to you as well. What respect do I expect from you I wonder! I always provide care for you so what! It is merely my faith which makes me to be here.”* (46 years old sister).

Love, patience, and knowledge of the problem were other coping strategies mentioned by the family caregivers as important for difficult situations. However, none of the caregivers had abandoned his/her relative but most showed signs of desperation.

## Discussion

This study has offered insight into the social and psychological problems of caring for mentally ill patients on families’ caregivers. Feelings and coping strategies experienced by family caregivers were revealed in the study. This study found that the main challenges faced by caregivers of mentally ill relatives were lack of social support, stigma, and conflict caused by the patients. Similar findings were reported in rural Ghana where caregivers reported financial difficulties, social exclusion, depression, and inadequate time for other social responsibilities as their main challenges [16].

The financial constraints found in this study corroborate findings from other studies that explored the relationship between mental illness and poverty [17]. In this study, it has been found that people with mental illness are often unable to generate income and that they often have to rely on the financial support of family members to meet basic living needs and to pay for any health expenditure associated with mental illness. Thus, family members may have to set aside a significant amount of their time to care for an ill family member. This can diminish caregivers’ chances to get or keep a job or earn income, which further increases the risk of poverty and poor mental health of the patient. This finding also shows similar financial burden as reported by a study conducted in Nigeria that explored relatives caring for schizophrenic patients [18]. However, our study did not explore the level of burden and its associations with caregivers’ educational level, age of patient, employment status of patients and global rating of difficulty in coping with caregiving.

In Mtwara region, the report from BasicNeeds in 2009 revealed that the shortage of psychotropic drugs resulted in substitution of patients’ usual medicine with alternative ones that were not as effective. In our research, the shortage of drugs was reported to decrease the credibility of health facilities, which are the main source of support for many families with mentally ill members. As such there is a need to improve mental health services including adequate provision of antipsychotic medication so that caregivers can continue getting them at the hospital free of charge as the policy stipulates.

Our findings demonstrate that there is a need for social support for the benefits of the family affected by mental illness. This unity helps to prevent stigma against the mentally ill patient in the family and the community at large. In the United State (US) research findings reports that families assume major roles in supporting relatives with mental illness [10]. The report in USA is inconsistent with these findings probably because of different cultural background of Tanzania and USA. However, education on the nature of mental illness can also be a problem, which calls for more emphasis on community awareness of mental illness in Tanzania. A qualitative study conducted in Thailand to explore the lived experience of Thai family caregivers found that extended family was a major source of support to caregivers physically, financially and emotionally [19]. This is not the case in this study because the caregiving was described to be occurring in a more nuclear family unit which resulted in decreased support to the family caregivers and may be due to the fact that the study was conducted in an urban setting. In essence, neighbours and the community are important components of social support. This research suggests that a strong social support system is critical for the improvement of mentally ill patients. However, a lack of this important component has hampered efforts to improve mentally ill patients’ condition and is the drawback to the psychosocial rehabilitation of chronic mentally ill patients. This argument is supported by findings from India which revealed several reasons for difficulties encountered by patients cared for at home [20]. The most important way to improve the social support system in Tanzania is to integrate mental health into the primary health care system [21]. In primary health care practice, family members with their mentally ill relative can access the health facilities easily and with low costs. Although mental health is addressed in the National Health policy of 2007, the pace of progress is too slow to cater to the increasing burden and demand of mental health care in the local setting. The government’s support is essential not only in improving primary mental health care but also in ensuring adequate provision of free treatment and free medication. Nevertheless, effort is needed by the government to increase the number of training institutions in order to create more mental health professionals.

Our study reported stigma as one of the biggest social challenges affecting families caring for patients with mental illness. Similar studies have shown that stigma may erode the morale of family caregivers and results in withdrawal of potential supporters [11, 15]. The report supports the finding in this study that some caregivers lose the support of their close relatives as well as other sources of social support after the onset of their relative’s mental illness. However, a longitudinal study is required to establish a causal relationship between the stigma and the loss of social support for families caring for relatives with mental illness. Due to the fact that participants were concerned about their caregiving obligations in this research, there is a significant need for education of the general population about the causes, presentation, and treatment of mental disorders. Although stigma and discrimination is a socially and culturally influenced phenomenon [3], increased education and understanding among the general population may decrease the stigma experienced by people who have a mental illness, their caregivers, and their families. Stigma is acknowledged globally as one of the major problems to the success of community mental healthcare and it prevents a person from being fully integrated into society. In Zambia, research found that stigma could be reduced if people with mental health problems were treated in primary health care settings rather than in mental or district hospitals [22]. In Tanzania, most health care facilities in the local setting have not yet incorporated mental health into their basic health care packages; this could aid in reducing stigma. Furthermore, research suggests that the media plays a pivotal role in the stigmatization of mental illness and therefore could also play a role in the de-stigmatization process [23]. Media should be effectively utilized to improve the general population’s understanding of mental disorders.

Family functioning has been affected by caring for patients with mental illness. There is change in the role of family members as the parents become involved or more consumed by patient’s needs. The patient has many needs which must be fulfilled by the caregivers at the same time that they must engage in daily tasks to earn an income. This increases fact increases the overall burden on the caregivers. A large quantitative study in Australia reported that higher levels of the burden of care were associated with lower levels of family functioning, which in turn were associated with higher levels of anxiety, depression and perceptions of poor health [24]. Our findings also demonstrated that there was a low level of family functioning that was associated with patient’s uncontrolled behaviour. The results of this research would further suggest that families affected by mental illness feel less cohesive and perceive themselves to be less connected to one another and less bonded as a unit than the normative family. In addition, the families of the mentally ill felt dissatisfied with the functioning of their family. This gives an impression that individual families should maintain their roles and relationships, thereby enabling the caregivers to maintain equilibrium. Any occurrence of mental illness of a family member which needs caring at home results in psychological and emotional disturbances for the whole family [25]. In relation to the conceptual approach of family caregiving [26], these family beliefs and relationships can be of great importance to understanding caregiving problems and any socio-cultural factors influencing family care of people with mental illness. However, it is also important to examine further and distinguish between the availability of social support resources and the actual use of that resource to strengthen family functioning.

Violence and threatening behaviour displayed by patients in this study would seem to be the result of insufficient symptom management. Proper management is of importance not only to the family caregiver but to all people who come into contact with the patient. This research has found that families are perplexed by violent patients. This is because many violent patients fail to see their behaviour as threatening and may actually perceive the family to be threatening. This threatening behaviour of patients comes at the cost of family caregivers who are often required to pay fines for destroyed property or pay compensation for injury caused by the patient. This phenomenon demoralizes the caregivers and further increases the stigma of mental illness. Information in regards to approach and management of violence are required when interacting with the mentally ill person.

Coping strategies are an important aspect of caring for a patient with mental illness. In this study caregivers have shown a narrow range of strategies for dealing with patients’ disruptive behaviours. Acceptance and praying are one of the most cited coping strategies. Some researchers have revealed different ways of coping with distressing situations including cognitive, behavioural and avoidance strategies [14, 27]. This is not the case in our study as most of them were engaged in caring activities, even though with difficulties.

A research study which specifically examined differences in coping strategies among caregivers of different age, ethnic, gender, and education groups found some evidence that older people were more religious than younger adults [27]. In their study, religious coping seemed to focus more on informal activities, especially informal beliefs, rather than on more formal religious activities. This is consistent with the findings in this study although we did not consider gender, age, ethnic and education background. Praying as one of the coping mechanism in our study, is similar to the findings from studies in Thai family caregivers [19]. However, there is a difference in religious beliefs in that Thai families were predominately Buddhist compared to mostly Christian and Muslim participants in Tanzania. In this research, both Christians and Muslims had developed a habit of praying as a way to deal with caregiving situation. Findings suggest that different consultation is sought as a way of finding solutions or treatments for their loved one. Some were advised to seek treatment from traditional healers but when they found no relief, they turned back to God and sought hospital services. Most were satisfied with the hospital treatment after individual effort to seek information concerning the illness of their loved ones (cognitive coping). Maladaptive coping strategies were not described although though some did shown signs of desperation in caring for their ill relatives as they were tired and did not know what to do to make their ill relative feel better.

### Limitation of the study

There are several limitations in this research. Firstly, the sample was too small with few participants in each FGD. This might have an effect in a normal interaction during the interview and consequently contributed to limited information. Secondly, the FGD groups were not homogenous which might have affected the freedom of expression among the participants.

Thirdly, only one primary caregiver and who spent most of the time with the patient was recruited. There could be variety in the types of problems associated with caregiving amongst different caregivers, due to different family roles and perceptions of caregiving. Finally, the study was hospital-based which means the findings are not generalizable to a community-based sample.

## Conclusion

Family caregiving for persons with mental illness has its advantages, yet it has multiple social and psychological challenges for both family caregivers and mental health professionals. Coping strategies and skills are important to the well-being of the caregiver and the patient.

Tackling of these problems needs a collaborative approach between health care providers and the government so that the needs of the caregiver and the family in general can be addressed.

Replication of the study with a larger sample based on age, gender, education background, occupation will be useful for further enriching the knowledge base of the phenomenon and in providing a foundation for future research in clinical practice.

### Ethics approval and consent to participate

Ethical clearance was obtained from the Muhimbili University of Health and Allied Sciences Ethics and Publication Committee. Further permission and approval was obtained from the Temeke Municipal Medical Officer in charge. Before the interview started, the informants were given a full explanation of the data collection procedure. Each participant was fully informed and understood the nature of the study and voluntarily agreed to participate. The written consent form was read, understood, and signed by the participants.

### Consent for publication

The participants were informed that the findings of the study would be published in different journals and that their names would not appear in the publications. The participants agreed and signed the consent form.

## Abbreviations

FGDs: Focus Group Discussions
MUHAS: Muhimbili University of Health and Allied Sciences
UBS: Uganda Bureau of Statistics
UNAIDS: The Joint United Nation Programme for HIV/AIDS
UNHS: Uganda National Health Services
WHO: World Health Organization
